# Understanding Cerebellar Liponeurocytomas: Case Report and Literature Review

**DOI:** 10.1155/2014/186826

**Published:** 2014-01-08

**Authors:** M. Y. Oudrhiri, N. Raouzi, I. El Kacemi, N. El Fatemi, R. Gana, M. R. Maaqili, F. Bellakhdar

**Affiliations:** Neurosurgery Department, Hopital Ibn Sina, P.O. Box 20503 Hay Riad, 10104 Rabat, Morocco

## Abstract

Cerebellar liponeurocytomas were recognized in the 2000 WHO 3rd edition of CNS tumors as a distinct grade I pathological entity, a tumor with a more favorable prognosis than medulloblastoma. But reports of long-term recurrences and some possible aggressive behavior led to an upgrade on the latest WHO 4th edition of CNS tumors. The case of a 64-year-old female patient is reported in this paper. More than 30 cases of this lately recognized pathological entity have been reported to date. The diagnostic, radiological, and pathological features associated with this tumor are discussed through a literature review.

## 1. Introduction 

The cerebellar liponeurocytoma was first included in the 2000 World Health Organisation's (WHO) 3rd edition of Central Nervous System (CNS) tumors' classification as a separate grade I entity. Thanks to a better recognition and longer follow-up, evidences of a possible tumor recurrence were growing. Rates of recurrences were estimated to 60% during follow-up, with some cases presenting even an aggressive behavior [1–3]. This way, the cerebellar liponeurocytoma was upgraded II on the 2007 WHO's 4th edition of CNS tumors' classification.

Through this case report we reviewed the literature for a better understanding of this pathology and the controversy around, with a listing of published cases of cerebellar liponeurocytoma.

## 2. A Case Report

A 64-year-old woman, with a 10-year history of Parkinson's disease, was admitted to our department for a 7-month clinical course of progressive worsening, right-sided weakness, and signs of increased intracranial pressure.

On examination, her consciousness was clear, and, beside the Parkinson's related tremor, she had a mild right hemiparesia (3/5) with a right Babinski sign and hyperreflexia on the right side. Ocular investigations showed bilateral papilledema and horizontal nystagmus.

MRI scan showed a 60 × 40 × 42 mm heterogeneous, enhancing lesion of the right cerebellum extending to the CPA (cerebellopontine angle), causing mass effect and mild hydrocephalous.

**Figure 1:**
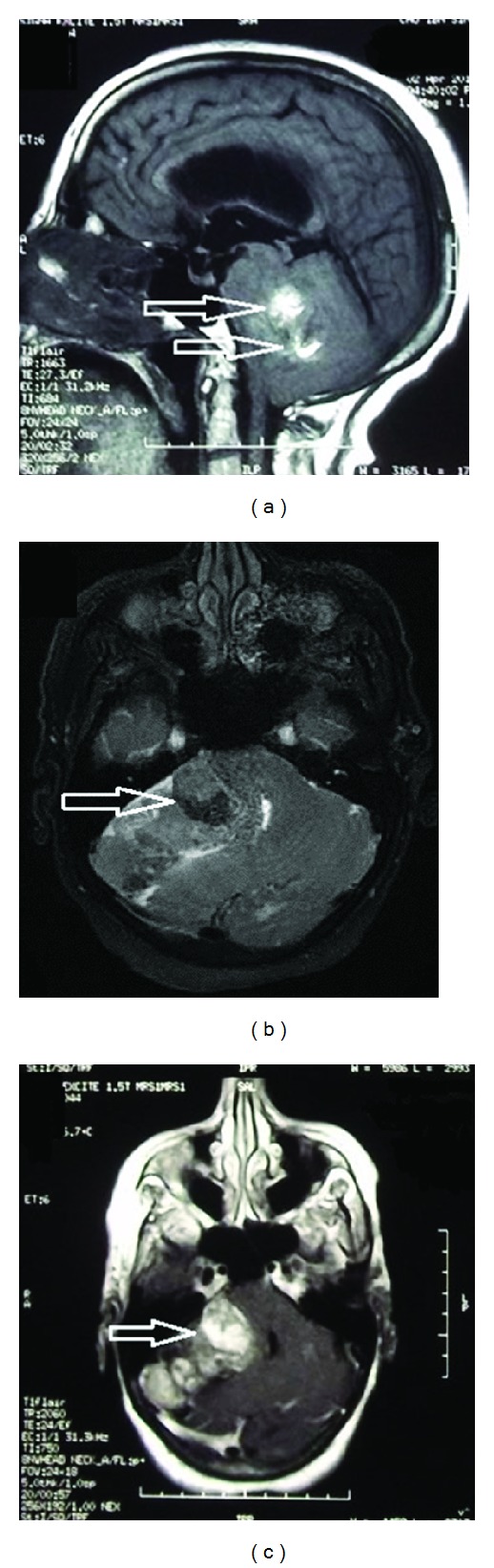
MRI of the brain in sagittal T1 (a), axial fat sat sequence (b), and axial T1 with gadolinium, respectively (c). Note the fat component (arrows in (a) and (c)) that turns hypointense after fat exclusion (arrow in (b)).

On T1-weighted images (Figure 1(a)) the mass was iso-intense to brain tissue, with sparse, hyperintense areas that turned hypointense on FAT-SAT sequences (Figure 1(b), arrow).

Irregular enhancement was noted after gadolinium administration (Figure 1(c)). There was no associated edema.

The patient underwent surgery through a retrosigmoid approach. It discovered a soft grey tumor, with yellowish areas, and globally well circumscribed. The anterior part of the tumor was encasing cranial nerves and was infiltrative toward the brainstem, which only allowed for near total removal. No cerebrospinal fluid shunt was decided.

**Figure 2:**
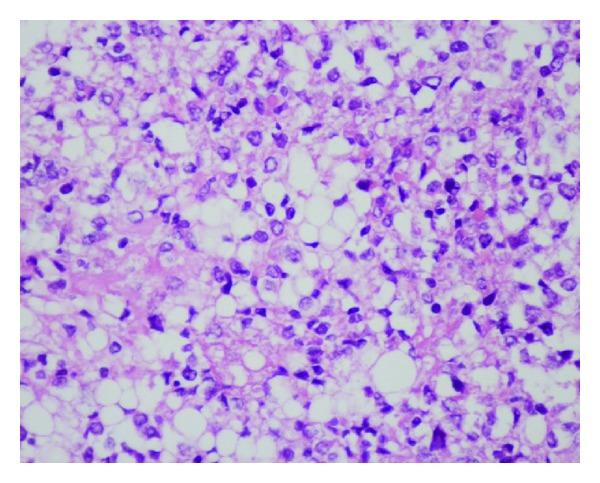
Histology results showing a typical field of liponeurocytoma; small round cells admixed with lipomatous cells. HES, original magnification ×10.

Pathological examination with immunohistochemical study revealed a tumor with astrocytic and neuronal differentiation (GFAP-NSE-synaptophysin positive) in which some focal areas of lipomatous differentiation were identified; the MIB-1 index was <1%; there was no cellular immaturity or necrosis, concluding on a cerebellar liponeurocytoma (WHO grade II) (Figure 2).

The postoperative course was free of complications and the patient was discharged from hospital to the postoperative care unit for physical rehabilitation. No adjuvant therapy was decided.

At two-year follow-up, the patient's condition was fair with no motor deficit and better autonomy (Karnofsky index improved from 40 to 70%).

## 3. Discussion

The cerebellar liponeurocytoma (LNC), as defined by the WHO, is a “rare, well differentiated neurocytic tumor of the cerebellum that arises in adults and typically shows focal or regional lipomatous differentiation. It has a low proliferative potential and a more favorable prognosis especially when compared to medulloblastoma (MDB), from which it needs to be distinguished” [4, 5]. In addition, the genetic profile of LNC is different from the medulloblastoma's but close to the central neurocytoma's profile (except for the presence of *TP53* mutation, absent in the later), which comforts the definition of cerebellar LNC as a separate pathological entity [6]. The association with a Parkinson disease was never reported; this may be incidental with no physiopathological correlation.

### 3.1. History

The cerebellar liponeurocytoma was first described in 1978, in a 44-year-old patient. Bechtel et al. named it “lipomatous medulloblastoma” and pointed to its better prognosis [7]. Since then, many other terms have been used to give name to this rare tumor: neurolipocytoma [8], medullocytoma [6], lipidized medulloblastoma [9], and lipomatous glioneurocytoma [10]. In 2000, the WHO adopted the term of “cerebellar liponeurocytoma” to replace all others.

### 3.2. Literature Review

Relying on a Pubmed research of Medline's database (using the keywords: lipidized medulloblastoma, medullocytoma, neurolipocytoma, and liponeurocytoma), we found 36 effective cases of pure cerebellar liponeurocytoma reported till now (Table 1). Controversy is rising on whether to consider cases exclusively located to the 4th ventricle and meeting the histological criteria of cerebellar liponeurocytoma as such or as central liponeurocytomas. Believing not and referring to the WHO's definition, the cases of Fung et al., Hsu et al., and Owler et al. were not included in our review [11–13]. To avoid this confusion, Chakraborti et al. suggests the designation “liponeurocytoma” rather than the restricted “cerebellar liponeurocytoma” to include other locations on the 4th and lateral ventricles [14].

Epidemiological analysis places cerebellar LNC as a tumor of adulthood (fourth and fifth decades), with mean age of 49 years (range from 32 to 74 years). The sex ratio, based on our review, shows a clear female prevalence (1 : 1.8). Giordana et al. and Sharma et al. reported cases of childhood onset, but these are more likely MDB with lipidized cells than lipomatous MDB [15, 16].

It is a tumor of the cerebellar hemispheres; location on the vermis is not unusual and so is the extension to the CPA, and was reported by many authors [17–19]. Two cases of supra-tentorial extension from cerebellar LNC were reported [19, 20]. Spinal extension to the C1-C2 space from cerebellar LNC was reported in two cases [18, 21]. One case of spinal lumbar metastasis occurring 11 years after initial diagnosis was reported [19].

On MRI scan, these tumors are globally heterogeneous, iso-, or hypointense on T1-weighted images, with areas of high signal intensity. These areas turn hypointense on fat suppression sequences, which may be very suggestive although there is no specific radiological behavior. Contrast enhancement is usually present and reported as irregular and heterogeneous; on T2-weighted images, the solid component is slightly hyperintense with focal pronounced hyperintense areas corresponding to fat. Some cystic components may be present, but usually no edema is reported [22].

One case of central tumor hemorrhage was reported on radiological findings [1].

### 3.3. Pathology and Genetics

Typically, cerebellar LNC is a glioneuronal tumor with lipidized neoplastic cells that resemble mature adipocytes. These are interspersed throughout the lesion. Areas of neuronal differentiation are usually positive for immunochemistry neuronal markers (synaptophysin; MAP-2). Reactivity for GFAP was also described [23–25]. No mitosis, necrosis, or vascular hyperplasia is usually seen.

Proliferation index, as measured by Ki-67/MIB-1, is usually low (<5%) in most published cases [1, 5, 6, 10, 17, 20, 23], which comforts the interpretation of the lesion as a benign one. However, many recent papers of separate cases reported relatively high rates of Ki-67 with cytopathologic atypia. These cases were associated with high rates of recurrences and a more aggressive behavior [3, 26–29]. Based on an analysis of relapse cases, some authors suggested the existence of different grades of malignancy of the LNC [3, 28].

To date, no specific genetic marker has been clearly identified. However, in their recently published paper, Anghileri et al. found a significantly positive overexpression of two possible markers: NEUROG1 and FABP4 [19]. Further studies may assess these findings.

### 3.4. Treatment

Optimum treatment modality is still a matter of debate. If all recommend surgery as the initial management, postoperative radiotherapy does not make the unanimity. In fact, the lack in understanding the tumor's biological behavior led to various therapeutic approaches.

The strong belief of it being a benign tumor often led surgeons not to choose adjuvant therapies whatever the extent of resection was. Moreover, the absence of histopathological patterns of malignancy comforted this attitude [27]. Further publications with long follow-up reported recurrence rates as high as 30–50% in the surgery groups with a median time to relapse of 10 years [3, 27, 28]. Limaiem et al. reported a rate of 31% based on an analysis of relapse cases [20].

The efficacy and the need for postoperative radiotherapy are still unclear. The scarcity of the tumor does not allow structured studies, and radiotherapy was used in many ways (EBRT; IORT) with different follow-up periods which makes literature very inhomogeneous. In fact, it seems not to prevent early recurrence [2]. The place of radiosurgery, given the low proliferative rate of these tumors, also needs to be established.

Management of recurrence is also debatable, but radiotherapy following reoperation seems to be more appropriate especially when secondary aggressive features are observed.

Our patient did not receive postoperative radiotherapy based on the belief that radiotherapy is to be a recurrence modality management after reoperation as for Cacciola et al. [27].

### 3.5. Survival

To date, the longest survival time recorded is of 18 years in two patients [17, 18]; both were treated with surgery only, even on relapse.

## 4. Conclusion

All this data analysis makes the prognosis and the evolution of this tumor still unpredictable. Its management is more debatable but all agree on a large surgical removal and a long follow-up. Adjuvant radiotherapy may be indicated if subtotal removal was performed or only on relapse after reoperation.

This paper represents an up-to-date literature review of all published cases of cerebellar liponeurocytoma.

## Figures and Tables

**Table 1:** Review of cases of cerebellar liponeurocytoma (including ours).

Author/year	Designation	Age/sex	Location	Treatment/adjuvant therapy	Proliferation index	Follow-up	Recurrence
Bechtel et al. 1978 [7]	Mixed mesenchymal and neuroectodermal tumor	44/M	LCH	Surgery	—	Died postop	—
Chimelli et al. 1991 [30]	Lipomatous differentiation in MDB	42/M	LCH	Surgery (GTR)	—	5 years 4 months	None
Davis et al. 1993 [9]	Lipidized MDB	49/F	Vermis	Surgery (STR)	—	5 years	None
		53/M	Vermis with bilateral extension	Surgery (GTR)/RT	—	Died at 6 months	—
Ellison et al. 1993 [8]	Neurolipocytoma	50/F	RCH	Surgery	<1%	4 years	None
Soylemezoglu et al. 1996 [17]	Lipomatous MDB	48/F	RCH	Surgery (STR)/RT	<1%	3.5 years	None
		59/F	LCH and CPA	Surgery (GTR)/RT	<3%	5 years	None
Giangaspero et al. 1996 [6]	Lipidized MDB	36/M	LCH	Surgery	<1%	15 years	1st R at 10 years, 2nd 5 years later (reoperation on each)
		37/M	LCH	Surgery	<1%	11 years	1st R at 10 years, 2nd one year later; died postop of ICH
		57/F	Vermis	Surgery/RT	<1%	2 years	None
Orlandi et al. 1997 [31]	Lipomatous MDB	55/M	Vermis and LCH	Surgery (GTR)	<1%	8 months	None
Alleyne et al. 1998 [10]	Lipomatous glioneurocytoma	66/M	RCH and CPA	Surgery (GTR)/RT	3.5%	—	—
Alkadhi et al. 2001 [18]	Cerebellar LNC	38/M	Vermis and RCH	Surgery (GTR)	Low	15 months	None
		53/M	LCH and CPA and C1 space	Surgery (GTR)	<5%	16 years	1st R at 12 years, 2nd 4 years later (reoperation on each)
Jackson et al. 2001 [1]	Cerebellar LNC	66/M	LCH with hemorrhage	Surgery (GTR)/RT	3%	6 months	—
Taddei et al. 2001 [26] Cacciola et al. 2002 [27] Buccoliero et al. 2005 [28] Pasquale et al. 2009 [3]	Cerebellar LNC	61/M	RCH	Surgery (GTR)	15% focally at onset; 20% at relapse	8 years	R at 3.5 years (surgery + IORT)
Montagna et al. 2002 [32]	Cerebellar LNC	53/F	RCH	Surgery (STR)/RT	<5%	4 years	None
Akhaddar et al. 2003 [33]	Cerebellar LNC	43/F	LCH	Surgery (GTR)/RT	<2%	16 months	None
El Shihabi et al. 2003 [34]	Lipomatous MDB	45/F	Multicentric: RCH, CPA, and LCH	Surgery (STR)/RT/CMT	<2.5%	3 years	None
Kachhara et al. 2003 [21]	LNC of the cerebellum	62/F	RCH and C1-2 space	Surgery (GTR)	—	—	—
Amina et al. 2003 [35]	Cerebellar LNC	38/F	LCH	Surgery	—	—	—
Jenkinson et al. 2003 [2]	Cerebellar LNC	51/F	RCH	Surgery (STR)/RT	<3%	15 months	1st R at 12 months, 2nd 3 months later (reoperation on each)
Val$\overset{´}{\text{e}}$ry et al. 2004 [36]	Cerebellar LNC	74/F	RCH	Surgery (GTR)/RT	5%	12 months	None
Aker et al. 2005 [23]	Cerebellar LNC	49/F	Vermis and 4th vt	Surgery (STR)/RT	<1%	Died at 19 months	None
Bazarbacha et al. 2005 [37]	Cerebellar LNC	38/F	Cerebellar	Surgery	—	—	—
Tatke and Singh 2005 [38]	Cerebellar LNC	32/F	Cerebellar	Surgery	—	—	—
Patel et al. 2009 [39]	Cerebellar LNC	40/F	RCH and CPA	Surgery (GTR)	—	3 years	None
Limaiem et al. 2009 [20]	Cerebellar LNC	42/M	RCH and vermis	Surgery (GTR)	2% at onset; <3% at recurrence	11 years	R at 10 years: reoperation
Ch$\hat{\text{a}}$tillon et al. 2009 [40]	Cerebellar LNC	42/F	RCH	Surgery (GTR)/RT	5 to 10%	3 years	None
Chakraborti et al. 2011 [14]	Cerebellar LNC	45/F	Midline	Surgery (GTR)	<1%	—	—
Anghileri et al. 2012 [19]	Cerebellar LNC	40/F	Vermis	Surgery (GTR)	1-2%	11 years	R at 8 years (no action)/spinal metastasis at 11 years: resection
		59/F	Vermis	Surgery (STR)	1-2%	10 years	R at 15 months: reoperation and radiosurgery
Guan et al. 2012 [22]	Cerebellar LNC	50/M	Cerebellar	Surgery	—	—	—
Chung et al. 2012 [29]	Cerebellar LNC	49/M	Vermis and 4th vt	Surgery (GTR)/RT	13.6%	12 months	—
Beizig et al. 2013 [41]	Cerebellar LNC	45/F	Vermis	Surgery (GTR)	—	18 months	None
Dey et al. 2013 [42]	Cerebellar LNC	30/F	LCH	Surgery (STR)	3%	6 months	—
Present case 2013	Cerebellar LNC	64/F	RCH and CPA	Surgery (STR)	<1%	2 years	None

LNC: liponeurocytoma; MDB: medulloblastoma; RCH: right cerebellar hemisphere; LCH: left cerebellar hemisphere; CPA: cerebellopontine angle; GTR: gross total removal; STR: subtotal removal; RT: radiotherapy; R: recurrence; ICH: intracerebral hemorrhage; IORT: intraoperative radiotherapy.
